# Historical perspective on ruthenium-catalyzed hydrogen transfer and survey of enantioselective hydrogen auto-transfer processes for the conversion of lower alcohols to higher alcohols

**DOI:** 10.1039/d2sc05621f

**Published:** 2022-10-26

**Authors:** Eliezer Ortiz, Jonathan Z. Shezaf, Weijia Shen, Michael J. Krische

**Affiliations:** Department of Chemistry, University of Texas at Austin, Welch Hall (A5300) 105 E 24th St. Austin TX 78712 USA mkrische@cm.utexas.edu

## Abstract

Ruthenium-catalyzed hydrogen auto-transfer reactions for the direct enantioselective conversion of lower alcohols to higher alcohols are surveyed. These processes enable completely atom-efficient carbonyl addition from alcohol proelectrophiles in the absence of premetalated reagents or metallic reductants. Applications in target-oriented synthesis are highlighted, and a brief historical perspective on ruthenium-catalyzed hydrogen transfer processes is given.

## Abstract 2

Ruthenium-catalyzed hydrogen auto-transfer reactions for the direct enantioselective conversion of lower alcohols to higher alcohols are surveyed. A brief historical perspective on ruthenium-catalyzed hydrogen transfer is given.

## Graphical abstract

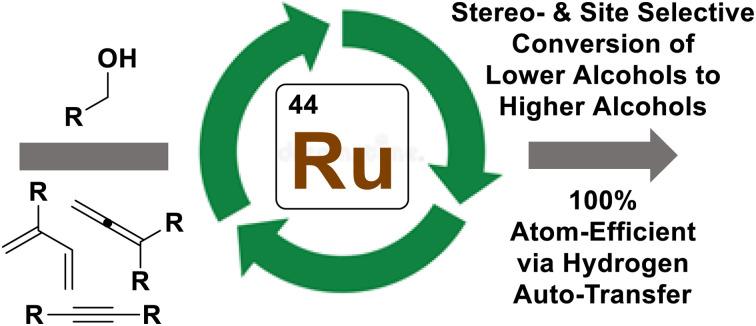

## I. Historical perspective

**Fig. 1:**
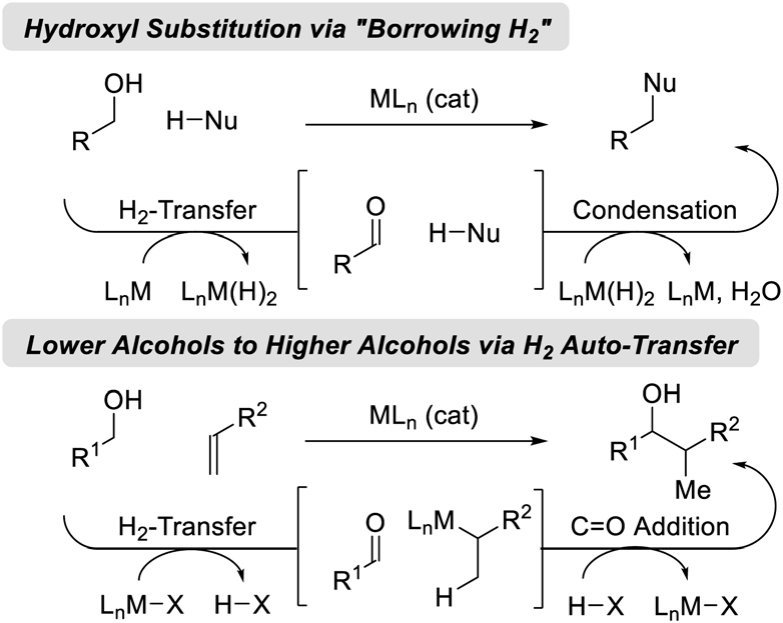
Two major classes of hydrogen auto-transfer processes.

Catalytic hydrogenation and transfer hydrogenation account for roughly 14% of reactions used to prepare small-molecule clinical candidates (GMP reactions),^1^ and it is estimated that hydrogenation catalysis will continue to expand.^2^ Beyond hydrogenation, a survey of >9 million patents demonstrate that carbonyl additions (alongside Suzuki couplings) are among the most widely utilized methods for C–C bond formation in the pharmaceutical industry.^3^ These data have inspired our laboratory to develop a family of metal-catalyzed hydrogen auto-transfer reactions^4^ that merge the characteristics of catalytic hydrogenation and carbonyl addition by exploiting the native reducing power of alcohols for the concomitant generation of organometallic nucleophiles (from π-unsaturated pronucleophiles) and carbonyl electrophiles. In this way, carbonyl addition is achieved from the alcohol oxidation level in the absence of stoichiometric organometallic reagents. Processes of this type convert lower alcohols to higher alcohols, and are distinct from so-called “borrowing hydrogen” reactions,^5^ which affect formal hydroxyl substitution (Fig. 1).

**Fig. 2:**
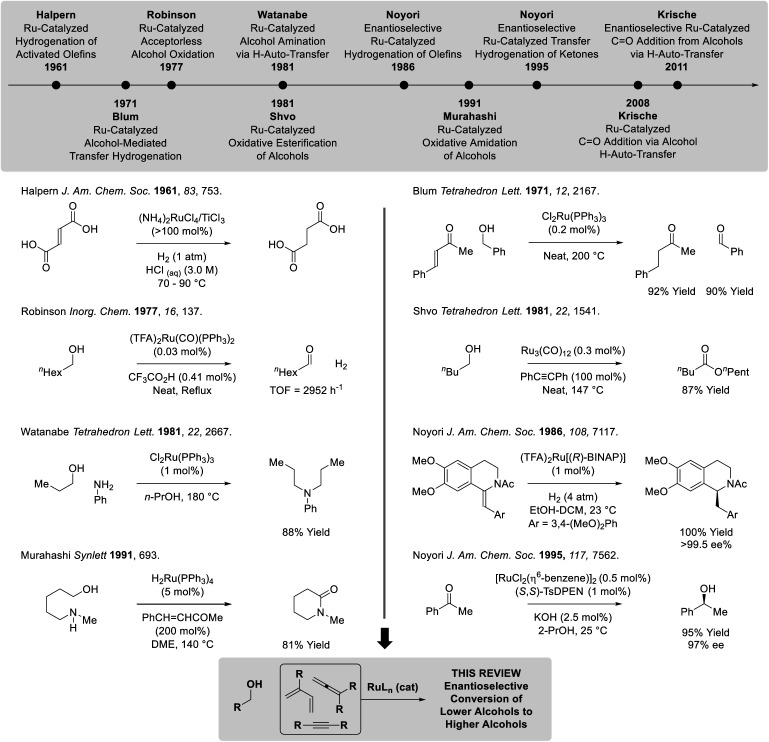
Selected milestones in homogeneous ruthenium-catalyzed hydrogen transfer.

Ruthenium(ii) complexes are octahedral d^6^ metal ions with unoccupied d_*x*^2^−*y*^2^_ orbitals, making them well-suited for alkoxide β-hydride elimination (Fig. 2). Indeed, beyond borrowing hydrogen processes,^5^ diverse transformations based on ruthenium(ii)-catalyzed alcohol dehydrogenation have been developed.^6^ Important milestones in ruthenium-catalyzed hydrogen transfer include the very first examples of alkene homogenous hydrogenation developed by Halpern (1961),^7^ ruthenium-catalyzed transfer hydrogenations reported by Blum (1971),^8^ so-called “acceptorless” alcohol dehydrogenations described by Robinson (1977),^9^ oxidative esterifications devised by Shvo and Murahashi (1981)^10*a*,*b*^ and related oxidative amidations (Murahashi 1991),^10*c*^ respectively, as well as borrowing hydrogen alcohol aminations described by Watanabe (1981).^11^ In a major advance, Noyori developed highly enantioselective ruthenium-catalyzed hydrogenations and transfer hydrogenations (1986 and 1995, respectively).^12^ Finally, ruthenium-catalyzed hydrogen auto-transfer reactions that transform lower alcohols to higher alcohols were developed in the present authors' laboratory (2008).^13,14^ Corresponding enantioselective ruthenium-catalyzed processes soon followed (2011) and, in recent years, have expanded to encompass diverse reaction types. In this monograph, enantioselective methods for conversion of lower alcohols to higher alcohols *via* ruthenium-catalyzed hydrogen auto-transfer are cataloged.^15^ For surveys on the broader topic of metal-catalyzed carbonyl reductive couplings, the reader is referred to the review literature.^16^

## II. Enantioselective reactions of alcohols with diene and allene pronucleophiles

**Scheme 1:**
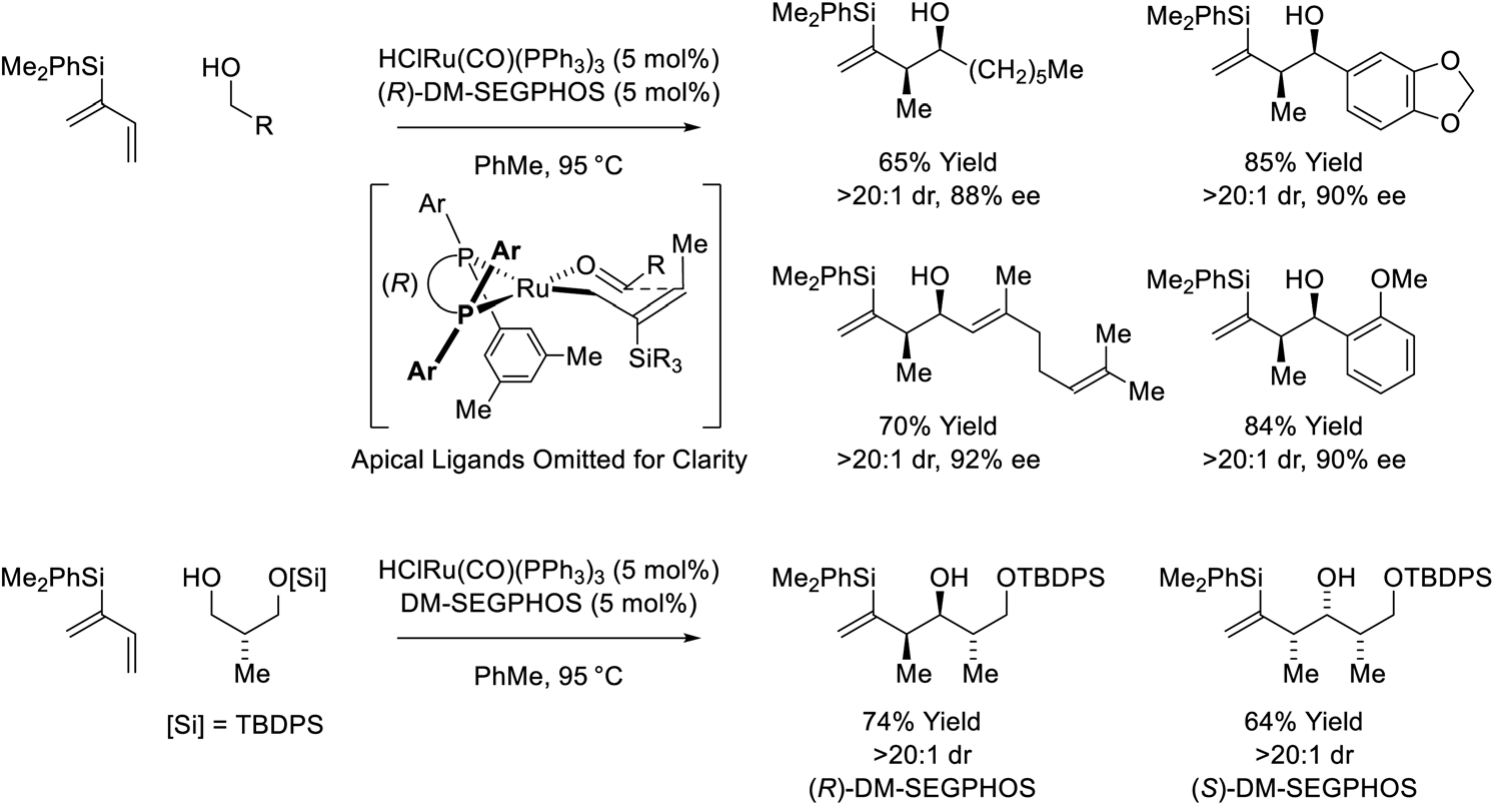
*syn*-Diastereo- and enantioselective silaprene-mediated crotylation of primary alcohols.

**Scheme 2:**
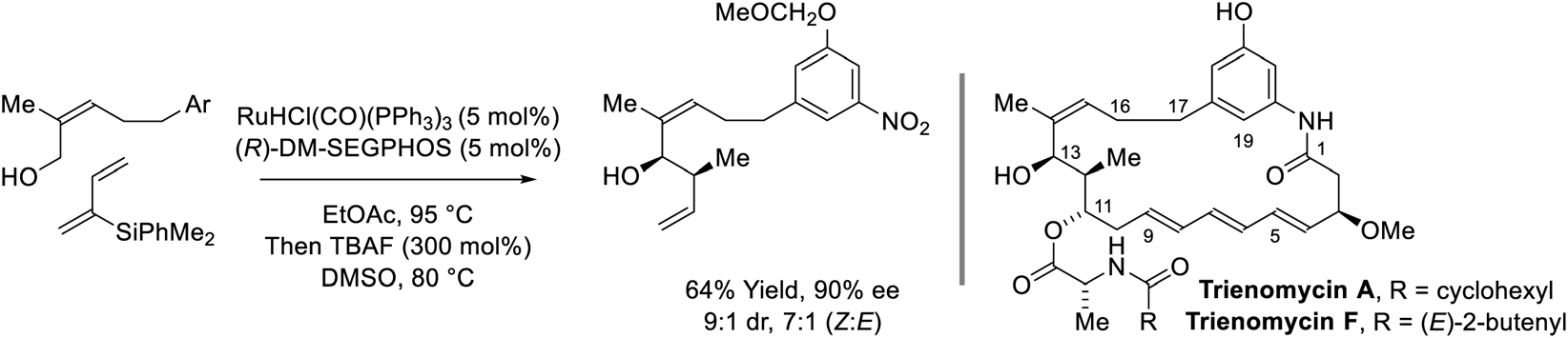
*syn*-Diastereo- and enantioselective silaprene-mediated crotylation of primary alcohols for the total synthesis of trienomycins A and F.

Following reports of racemic diene-alcohol C–C couplings *via* hydrogen auto-transfer,^13,17^ the first enantioselective ruthenium-catalyzed transformations of this type were developed (Scheme 1).^18*a*^ Using a chiral ruthenium catalyst modified by (*R*)-DM-SEGPHOS, hydrogen transfer from primary alcohols to 2-trialkylsilyl-butadienes (“silaprenes”) generates crotylruthenium–aldehyde pairs that combine to form branched secondary homoallylic alcohols with high levels of enantiomeric enrichment. Allylic 1,2-strain imposed by the trialkylsilyl moiety defines alkene geometry of the transient σ-crotylruthenium nucleophile, which participates in stereospecific aldehyde addition through a closed transition structure to deliver the *syn*-diastereomers. Brimble exploited enantiomeric ruthenium catalysts in couplings of silaprene with the indicated chiral α-stereogenic alcohol derived from the Roche ester, enabling access to the *syn*,*anti*- or *syn*,*syn*-diketide stereotriads with good levels of catalyst-directed diastereocontrol.^19^ The silaprene-mediated conversion of lower alcohols to higher branched alcohols was utilized in total syntheses of trienomycins A and F (Scheme 2)^21*a*^ and soraphen A (not shown).^21*b*^

**Scheme 3:**
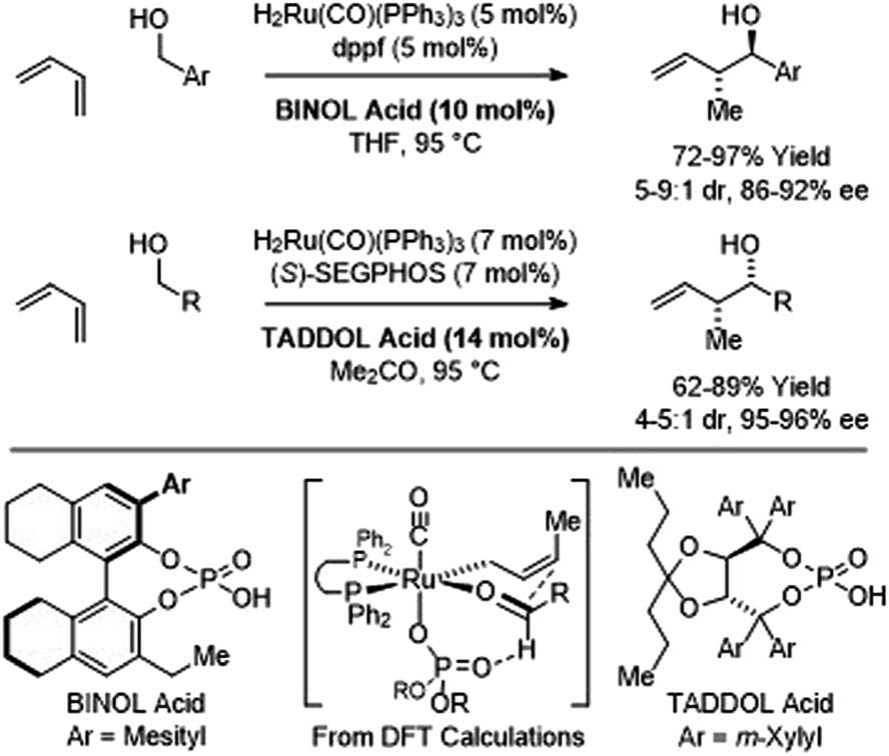
Chiral counterion-directed *anti*- and *syn*-diastereo- and enantioselective butadiene-mediated crotylations of primary alcohols.

**Scheme 4:**
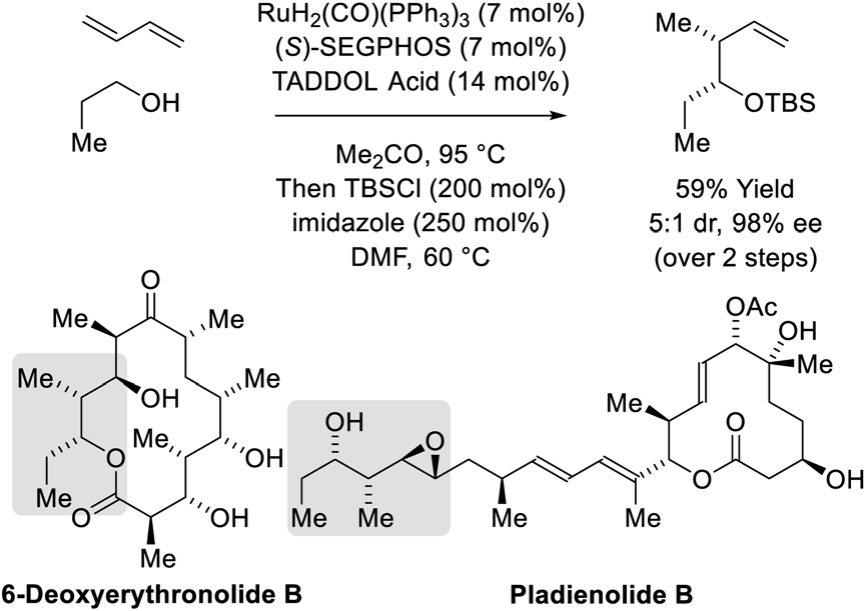
*syn*-Diastereo- and enantioselective butadiene-mediated crotylation of primary alcohols in the total synthesis of 6-deoxyerythronolide B and pladienolide B.

In C–C couplings of primary alcohols with the parent diene, butadiene (an abundant petrochemical feedstock), high levels of stereoselectivity are more difficult to achieve due to the challenge of controlling the geometry of the intervening σ-crotylruthenium nucleophile. Eventually, it was found that good levels of *anti*-diastereoselectivity and enantioselectivity could be obtained using ruthenium catalysts bound by chiral BINOL-derived phosphate counterions (Scheme 3).^18*b*^ However, these processes were restricted to benzylic alcohols. Interestingly, using the corresponding chiral tartaric acid-derived phosphate counterion in combination with (*S*)-SEGPHOS, *syn*-diastereo- and enantioselective crotylations could be achieved (Scheme 3).^18*c*^ It was postulated that the more Lewis basic tartaric acid-derived phosphate counterion forms a contact ion pair with ruthenium, retarding the rate of isomerization of the kinetic (*Z*)-σ-crotylruthenium haptomer (formed by hydroruthenation of the s-*cis* conformer of butadiene) with respect to carbonyl addition. Computational studies suggest the transition state for aldehyde addition leading to the *syn*-diastereomer is stabilized by a formyl hydrogen bond.^20,22^ The *syn*-diastereoselective butadiene-mediated conversion of lower alcohols to higher branched alcohols was utilized in total syntheses of 6-deoxyerythronolide B^23^ and pladienolide B (Scheme 4).^24^

**Scheme 5:**
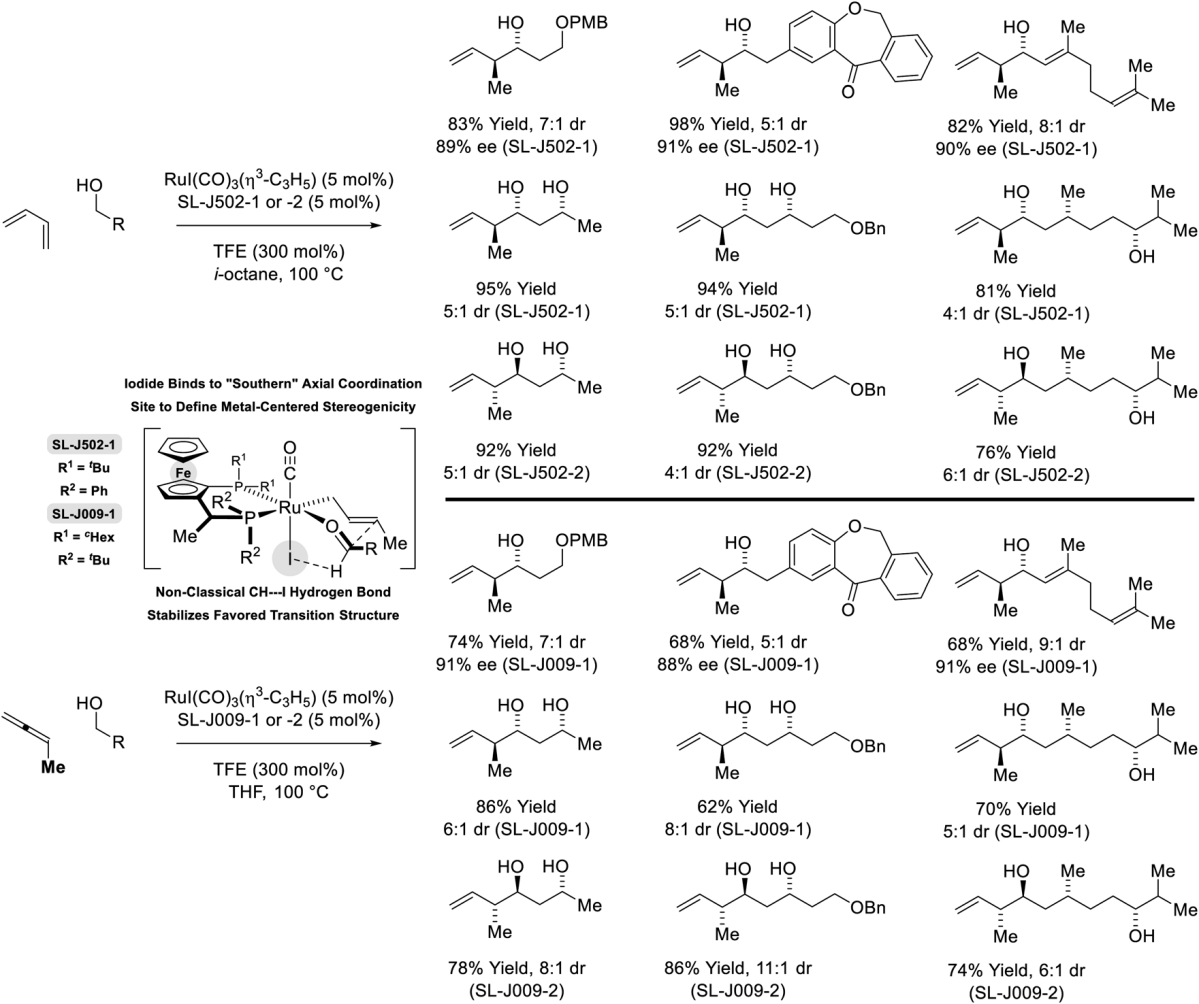
*anti*-Diastereo- and enantioselective crotylation of primary alcohols by the C4 feedstocks 1,2- and 1,3-butadiene.

In more recent work, it was found that iodide-bound ruthenium-JOSIPHOS complexes catalyze the coupling of primary alcohols with butadiene to generate diastereo- and enantiomerically enriched products of *anti*-crotylation (Scheme 5).^18*d*^ This catalytic system displays broad scope and is of greater practicality, as it does not require chiral counterions and the JOSIPHOS ligand is commercially available. One remarkable feature of the ruthenium-JOSIPHOS-catalyzed primary alcohol-butadiene C–C coupling relates to the ability to affect C–C coupling of primary alcohols in the presence of unprotected secondary alcohols without the need for hydroxyl protecting groups, which is attributed to the relatively rapid kinetics of primary alcohol dehydrogenation. As supported by experimental and computational studies, iodide-bound ruthenium-JOSIPHOS complexes (unlike the corresponding chloride or bromide complexes) enforce high levels of enantioselectivity by defining the stereogenic center at ruthenium and through formation of a formyl CH⋯I hydrogen-bond that stabilizes the favored transition state for carbonyl addition.^22^ Nearly identical reaction conditions were effective in couplings of primary alcohols with methylallene to form a duplicate set of diastereo- and enantiomerically enriched products. These processes represent an alternative to the longstanding use of discrete allylmetal reagents in enantioselective carbonyl crotylation.^25^

**Scheme 6:**
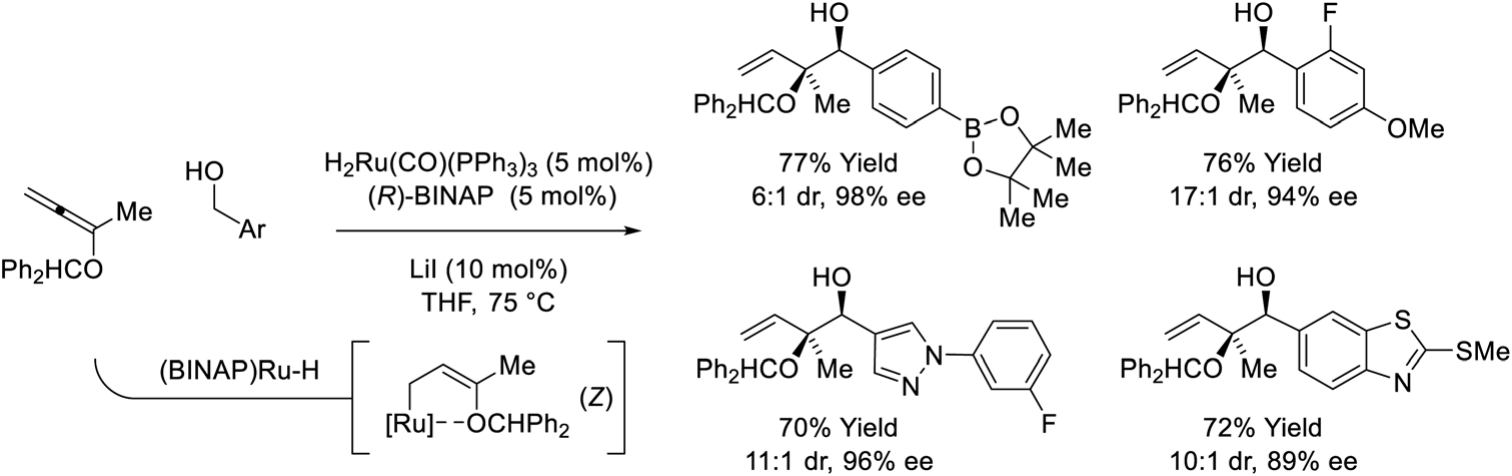
*syn*-Diastereo- and enantioselective alkoxyallene-mediated conversion of primary alcohols to *syn*–*sec*,*tert*-homoallylic diols.

Ruthenium-BINAP-catalyzed couplings of primary benzylic alcohols with the indicated 1,1-disubstituted alkoxyallene enables formation of enantiomerically enriched *syn*–*sec*,*tert*-diols (Scheme 6).^26^ Unusual *syn*-diastereoselectivity is attributed to internal chelation of the benzhydryl oxygen to ruthenium, which directs formation of (*Z*)-σ-(alkoxy)allylruthenium isomers that participate in stereospecific carbonyl addition through chair-like transition structures. Low conversion was observed in attempted reactions of aliphatic alcohols, however, 2-propanol-mediated reductive couplings of alkyl-substituted aldehydes were achieved in good yield (not shown).

## III. Enantioselective reactions of alcohols with alkyne pronucleophiles

**Scheme 7:**
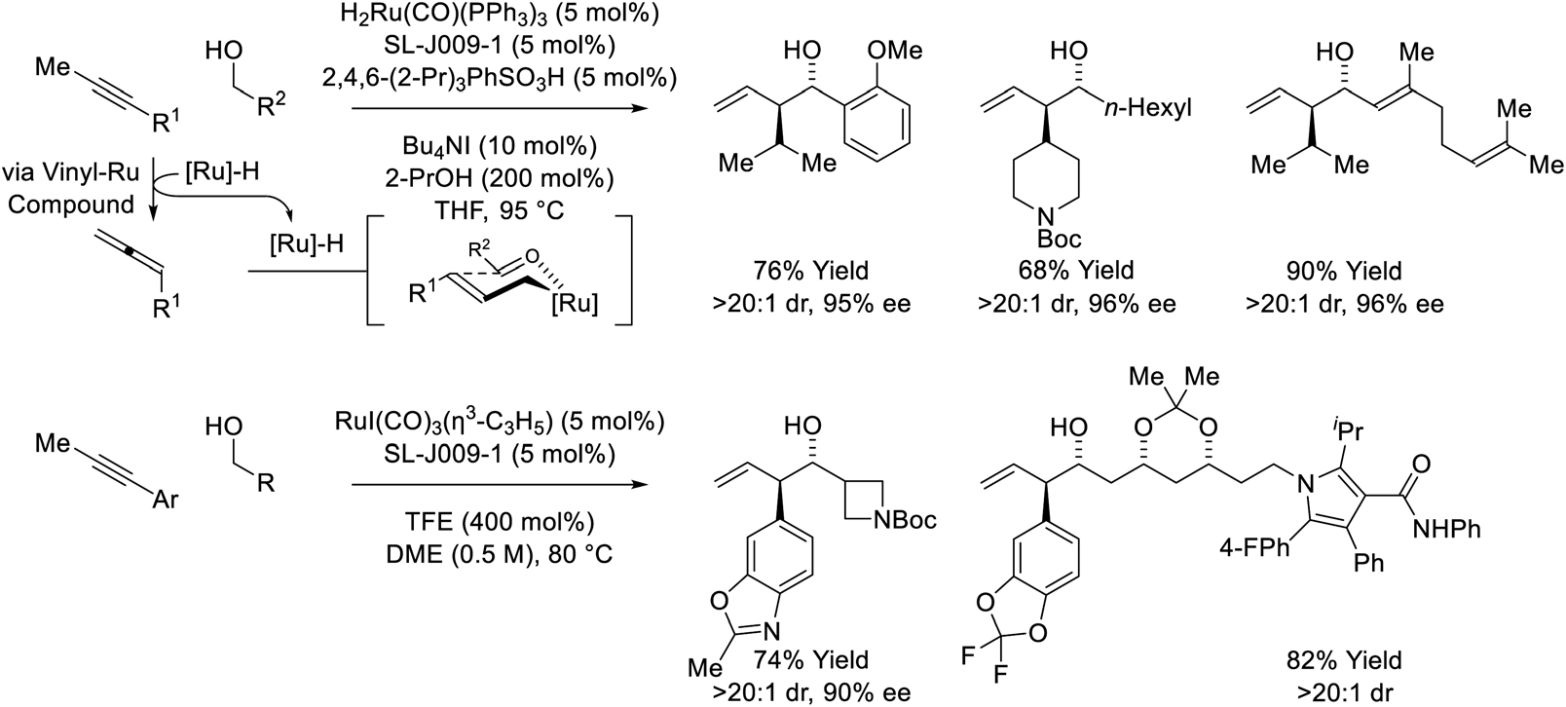
*anti*-Diastereo- and enantioselective alkyne-mediated conversion of primary alcohols to branched homoallylic secondary alcohols.

The first reported use of alkyne pronucleophiles in the enantioselective ruthenium-catalyzed conversion of lower alcohols to higher alcohols takes advantage of a dual catalytic cycle in which alkyne-to-allene isomerization (*via* hydrometallation-β-hydride elimination) is followed by allene-mediated carbonyl allylation to deliver branched homoallylic secondary alcohols (Scheme 7).^27*a*^ In these processes, iodide-bound ruthenium-JOSIPHOS catalysts are generated *in situ* through the acid–base reaction of H_2_Ru(CO)(PPh_3_)_3_ with 2,4,6-(2-Pr)_3_PhSO_3_H, followed by substitution of the sulfonate counterion with iodide. Chloride and bromide-containing catalysts were inferior. As previously indicated (*vide supra*, Scheme 5), iodide defines the stereogenic center at ruthenium and stabilizes the favored transition state for carbonyl addition through formyl CH⋯I hydrogen-bonding with the transient aldehyde. Several improvements to this catalytic system were subsequently made. Replacement of 2,4,6-(^*i*^Pr)_3_PhSO_3_H with 4-NO_2_PhSO_3_H resulted in >50% increase in isolated yield in reactions of 1-aryl-1-propynes, although catalyst loadings of 10 mol% remained necessary (not shown).^27*b*^ The use of the iodide-containing precatalyst RuI(CO)_3_(η^3^-C_3_H_5_) in combination with trifluoroethanol (TFE) provided the most effective catalytic system for the reaction of primary alcohols with 1-aryl-1-propynes to form products of carbonyl *anti*-(α-aryl)allylation.^27*c*^ In this process, TFE catalyses exchange of the homoallylic ruthenium alkoxide with reactant alcohol, mitigating issues of product inhibition.

**Scheme 8:**
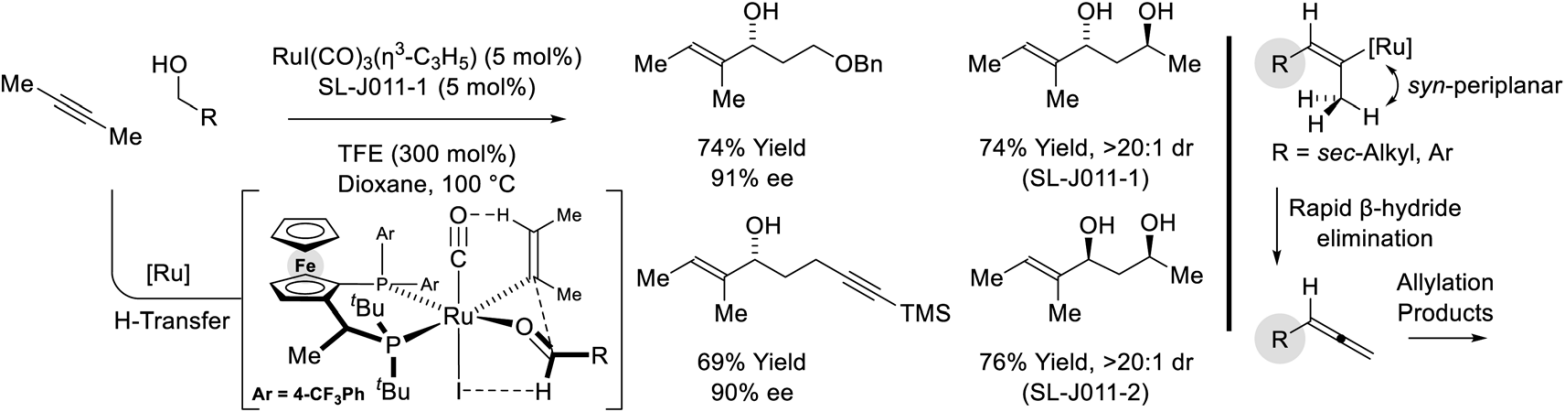
Enantioselective conversion of primary alcohols and 2-butyne to secondary allylic alcohols.

Remarkably, when the latter conditions are applied to 2-butyne, alkyne-to-allene isomerization is interrupted at the stage of intermediate vinylruthenium species *via* carbonyl addition to form products of alkenylation (Scheme 8).^28,29^ This divergent reactivity suggests acetylenic substituents larger than methyl (*i.e.* aryl or *sec*-alkyl) stabilize a *syn*-periplanar conformation of the carbon–ruthenium and propargylic C–H bonds, reducing the entropy of activation for β-hydride elimination to accelerate allene formation. The conversion of 2-butyne and primary alcohols to secondary allylic alcohols is highly chemoselective and proceeds in the presence of silyl-substituted alkynes and unprotected secondary alcohols. The indicated stereochemical model invokes a formyl CH⋯I hydrogen-bond^22,27*c*^ and a ruthenium CH···O≡C[Ru] hydrogen-bond^30^ in the favored transition state for carbonyl addition.

**Scheme 9:**
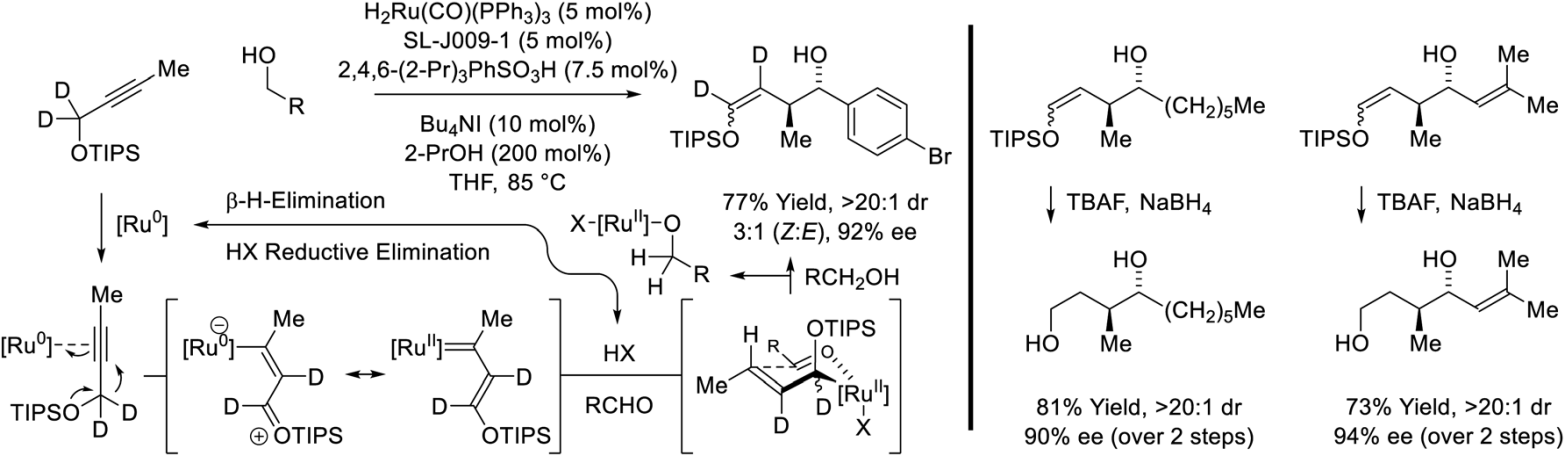
*anti*-Diastereo- and enantioselective conversion of primary alcohols and trialkylsilyl propargyl ethers to branched secondary alcohols.

Yet another mode of reactivity is observed in connection with the couplings of trialkylsilyl propargyl ethers and primary alcohols (Scheme 9).^31^ Under conditions essentially identical to those used in the coupling *sec*-alkyl propynes (*vide supra*, Scheme 7),^27*a*^ alkyne-to-allene isomerization is not observed. Rather, as corroborated by deuterium labeling studies, alkyne coordination by zero-valent ruthenium triggers a 1,2-hydride shift to form a vinyl carbene, which upon protonation, generates a siloxy-π-allylruthenium nucleophile. Aldehyde addition by way of the indicated σ-allylruthenium haptomer delivers branched homoallylic alcohols that incorporate enol silyl ethers. Silyl deprotection–reduction of the crude reaction products provides the corresponding 1,4-diols with excellent control of regio-, diastereo- and enantioselectivity.

## IV. Enantioselective reactions of alcohols with enyne pronucleophiles

**Scheme 10:**
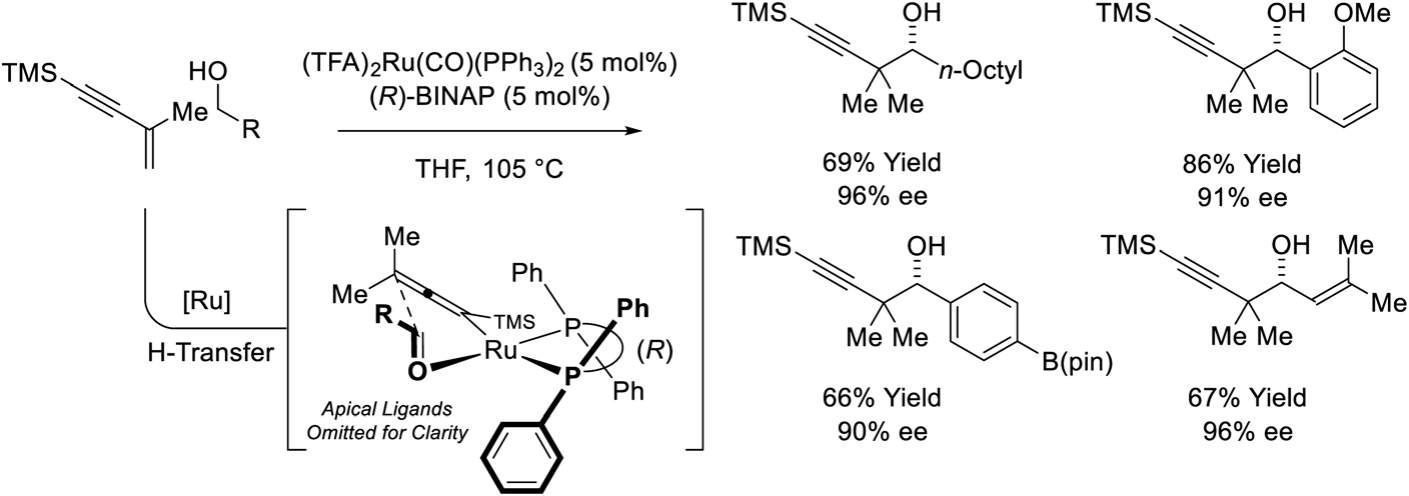
Enantioselective conversion of primary alcohols and 2-butyne to secondary allylic alcohols.

The first examples of enyne-mediated carbonyl propargylation were reported from the present authors laboratory.^32–34^ The first enantioselective ruthenium-catalyzed conversion of 1,3-enynes and primary alcohols to form homopropargylic secondary alcohol was achieved using the chiral ruthenium complex assembled from (TFA)_2_Ru(CO)(PPh_3_)_2_ and (*R*)-BINAP (Scheme 10).^32*c*^ The resulting secondary homopropargyl alcohols bearing *gem*-dimethyl groups are formed with excellent levels of enantiomeric enrichment. In this process, hydrogen transfer from the primary alcohol to the 1,3-enyne generates a prochiral allenylruthenium nucleophile, which engages the transient aldehyde in carbonyl addition by way of the indicated closed transition structure. In support of this mechanism, the reaction of HClRu(CO)(PPh_3_)_3_ with 1,3-enynes to form an isolable σ-allenylruthenium complex characterized by single crystal X-ray diffraction has been described.^35^ This protocol represents an alternative to the longstanding use of discrete allenylmetal reagents in enantioselective carbonyl propargylation.^36^

## V. Conclusion and future outlook

Ruthenium complexes are the prototypical hydrogen transfer catalysts and they continue to facilitate an ever-increasing array of chemical processes. As described in this perspective article, a new reactivity mode in the field of ruthenium catalysis is represented by the emerging class of chemical transformations in which alcohol dehydrogenation triggers reductive generation of aldehyde-organometal pairs that combine to affect byproduct-free carbonyl addition. Such reactions enable direct conversion of lower alcohols to higher alcohols and allow abundant π-unsaturated feedstocks to serve as surrogates to stoichiometric organometallic reagents. Discovered and developed wholly in the present authors laboratory, processes of this type raise numerous other possibilities. Beyond continued efforts to adapt this concept to other types of carbonyl additions (alkylations,^37^ vinylations,^38^ alkynylations, and arylation), methods for the direct conversion of lower amine to higher amines (hydroaminoalkylation)^39^ represents an important challenge that merits further attention. It is the authors' hope that the present state of the art summary will expedite progress toward this and other challenges in this growing area of research.

## Conflicts of interest

There are no conflicts to declare.

## Supplementary Material
